# Caring for people with dementia during the COVID-19 pandemic: a systematic review

**DOI:** 10.1590/1980-5764-DN-2023-0123

**Published:** 2024-10-25

**Authors:** Juan Jesús Robles-García, José Ángel Martínez-López

**Affiliations:** 1 Universidad de Murcia, Facultad de Trabajo Social, Departamento de Trabajo Social y Servicios Sociales, Murcia, España.

**Keywords:** Dementia, Cognitive Dysfunction, Pandemics, COVID-19, Demência, Disfunção Cognitiva, Pandemias, COVID-19

## Abstract

Human history has been linked to numerous risks to survival, with pandemics being a constant in human life. People suffering from dementia experienced greater difficulties accessing healthcare and treatment during the COVID-19 pandemic.

**Objective:**

To identify how the COVID-19 pandemic influenced care for people with dementia or cognitive impairment.

**Methods:**

This research work is a systematic review conducted with a literature search in four databases such as Web of Science, Scopus, EBSCOHost, Cochrane Library, and ProQuest, following the methodological proposals of the Preferred Reporting Items for Systematic reviews and Meta-Analyses (PRISMA) guide.

**Results:**

The bibliographic research in the different databases offered a total of 561 records, of which 23 were chosen to elaborate the results. The main results were the increasing cognitive impairment and psychosocial consequences of social distancing during the pandemic, including sadness, helplessness, and abandonment in patients or feelings of burnout and overload in caregivers and health professionals.

**Conclusion:**

The COVID-19 placed patients with dementia in the background. During the pandemic, attention was focused primarily on emergencies and not so much on the monitoring of chronic diseases, which also caused psycho-emotional and social worsening.

## RESUMO

A história da humanidade tem sido associada a inúmeros riscos à sobrevivência, sendo as pandemias uma constante na vida humana. As pessoas que sofrem de demência tiveram mais dificuldades de acesso a cuidados e tratamentos de saúde durante a pandemia de COVID-19.

**Objetivo:**

Identificar como a pandemia de COVID-19 influenciou o atendimento a pessoas com demência ou comprometimento cognitivo.

**Métodos:**

Este trabalho de pesquisa é uma revisão sistemática realizada com uma pesquisa bibliográfica em quatro bases de dados, como Web of Science, Scopus, EBSCOHost, Cochrane Library e ProQuest, seguindo as propostas metodológicas do guia *Preferred Reporting Items for Systematic reviews and Meta-Analyses* (PRISMA).

**Resultados:**

A pesquisa bibliográfica nos diferentes bancos de dados ofereceu um total de 561 registros, dos quais 23 foram escolhidos para a elaboração dos resultados. Os principais resultados foram o aumento do comprometimento cognitivo e as consequências psicossociais do distanciamento social durante a pandemia, incluindo tristeza, desamparo e abandono para os pacientes ou sentimentos de esgotamento e sobrecarga para os cuidadores e profissionais de saúde.

**Conclusão:**

A COVID-19 fez com que os pacientes com demência ficassem em segundo plano. Durante a pandemia, a atenção se concentrou principalmente nas emergências e não tanto no monitoramento de doenças crônicas, o que também provocou uma piora psicoemocional e social.

## INTRODUCTION

Dementia is defined as a loss of brain function that can affect different areas of our body, such as memory loss or impaired thinking and speech. It can also affect motor function (slowing of movement and loss of flexibility), and lead to behavioral disorders, or impaired judgment. It is an irreversible disease in most types as it is considered degenerative; on most occasions, the clinical condition only worsens, and there is no possibility of reversing it^1^. At the pathophysiological level, dementia usually occurs due to the accumulation of specific abnormal proteins in neuronal regions, glia, and extracellular compartments, or in the most defenseless areas of the brain.

Human history has been associated with numerous threats to survival, with pandemics being a constant in human life; from striking cases such as the Black Death in 1346 or the Spanish flu in 1918, the Influenza A in 2009 or the Ebola outbreak in 2014, to the arrival of SARS CoV-2 in 2019.

It is estimated that the coronavirus (COVID-19) could have started its first contagion almost at the end of 2019, but it was not until early 2020 that it began to spread worldwide. It is a contagious disease, transmitted through the SARS-CoV-2 respiratory virus via person-to-person respiratory droplets, which led to a massive contagion of the population due to the ease of human-to-human transmission, to the point of being considered a global pandemic^2^. Social restrictions led to a greater sense of loneliness and carelessness for dementia people; and due to this absence of care by health professionals, in most cases, a single family member was responsible for the assistance of the person with dementia, causing overload, anxiety, and physical and psychological weakening^3^.

In order to correct this lack of health services, telemedicine was implemented globally and widely, to monitor the patient through telephone medical consultations or, on some occasions, when required, through video calls. It represented a significant change at the socio-healthcare level that facilitated healthcare for the population without physical contact in order to curb the spread of COVID-19; on the other hand, it meant a break in the routine of dementia patients who were used to go for consultations which was positive for their clinical status^4^.

One of the main challenges for the population during the pandemic was the psychological and emotional support due to the serious impact of confinement, which increased vulnerability during the disease process. The numerous deaths caused by the COVID-19 pandemic provoked great concern and fear in the population due to the death of family members and loved ones, causing significant emotional distress. The changes caused by physical restrictions were considered another major challenge during this pandemic, involving a drastic change in the daily life of the entire population, leading to a distancing between loved ones and family^5^. In addition, Pinazo-Hernandis showed the persistence of social problems, such as loneliness and isolation in the elderly, already existing prior to the pandemic, which increased with the onset of the pandemic^6^.

Another point that arose as a consequence of the pandemic and which directly influenced dementia people was burnout syndrome, present in healthcare staff and caregivers of these patients. This is a professional term characterized by low personal fulfillment in a setting where levels of emotional exhaustion and depersonalization are increased due to decompensation in adapting to specific adverse events; in this case, the COVID-19 pandemic caused feelings of prolonged stress^7^. People with dementia were one of the most affected groups in this period of social restriction. Care for dementia patients was severely restricted as they were considered a fragile population and highly vulnerable to COVID-19 contagion^7^. That is why they faced great challenges in this hostile era, including the deficit in healthcare. Articles such as those by Wang and Mariani emphasized the feelings of loneliness and the unmet needs of the health system, which collapsed and lacked sufficient resources to reach these people^8,9^. Besides, the study by Aranda et al.^10^ mentioned the economic difficulties in reaching the dementia population as well as differences in care according to the patient’s region or race, which was also supported by Suemoto et al.^11^

## METHODS

This review article aimed to identify how the COVID-19 pandemic influenced the care for people with dementia or cognitive impairment, describing the psychological and social consequences for these people and their families, as well as the methods of approach from the health systems. This review was carried out following the criteria for writing systematic reviews according to the Preferred Reporting Items for Systematic reviews and Meta-Analyses (PRISMA) guidelines. In this work, no software was used for the systematization and analysis of the information.

Concerning the eligibility criteria, the studies taken into account for this review were those aimed at providing information on proposals for action for the care of people with dementia during the COVID-19 pandemic, both at home and in institutions. In addition, any study offering intervention programs or strategies for action in the care of dementia patients during the pandemic, such as protocols or intervention measures, was also collected. We also considered relevant all those studies that showed a perspective on care, both by professionals and patients themselves or their carers, thus providing a holistic view of the social phenomenon studied. Moreover, studies on fear and/or concerns about the availability of external care in a pandemic situation and burnout among healthcare workers and caregivers were also included.

No filtering about the type of study was applied for selection, and studies could be either qualitative or quantitative in nature. Articles were searched without any filter on language or availability as well.

Descriptive and comparative studies characterized as follows were excluded from the selection for this review:

- Care programs in the care of people with dementia during the pandemic were not mentioned;
- Programs that did not report on adverse psychological manifestations due to the pandemic and lack of care;
- Reports that did not show new approaches;
- Studies on book or dissertation reviews; and
- Newspaper article format.

The literature search strategy used in this paper focused on collecting the results of articles from the last three years in which the COVID-19 pandemic had a significant impact on the current population. With this in mind, and to carry out an exhaustive search to identify, retrieve, and code most of the studies addressing the issues outlined above, a search was launched in the databases ProQuest, Web of Science, Scopus, Cochrane Library, and EBSCOHost on November 14, 2021. These databases were chosen because they included a large part of the rest of the available journals, offering more global information.

Once the studies were selected to form part of this work, their possible biases were analyzed (1)^12-34^. To this end, the main recommendations for reading the Equator Network Reporting Guidelines, specific to each study — Consolidation Standards of Reporting Trials (CONSORT), Strengthening the Reporting of Observational Studies in Epidemiology (STROBE), etc. were used.

## RESULTS

**Figure 1:**
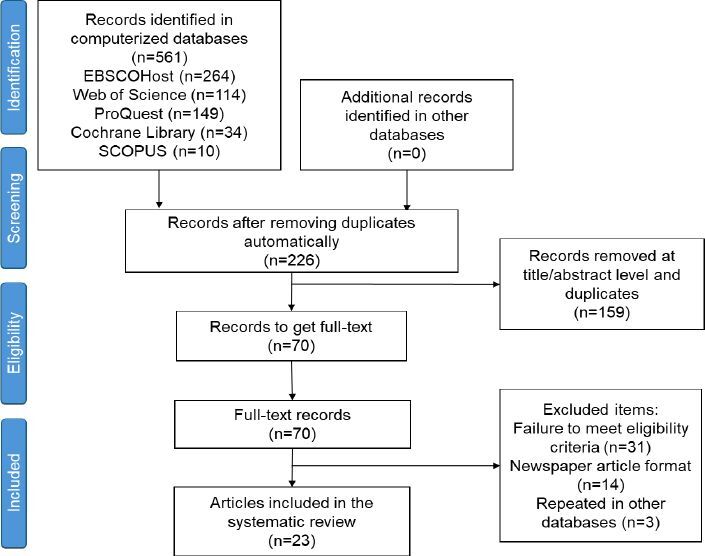
Flowchart of the study selection process.

Figure 1 shows the selection process of the studies included in the analysis. This literature search, launched between November 2021 and February 2022 in the different databases selected (EBSCOHost, Web of Science, Scopus, ProQuest, and Cochrane Library) produced 561 records. Of these, 345 were automatically excluded as they were duplicated between databases. Following the removal of duplicates, 216 studies were collected. Titles and abstracts were read to select, in a more generic way and according to the eligibility criteria, the studies that would later be searched for full text, with 60 chosen. Subsequently, we checked and obtained those studies that were available in full text.

Once the full-text reading was completed, 23 studies were found that met the established eligibility criteria and were finally included in this systematic review.

The studies finally chosen for the content of the results were divided into four variables:

- Studies that addressed the shortage of healthcare staff and the concern of patients and their carers about the need to seek healthcare services or about health service overload and the consequences thereof in patient care (14 of the 23 included studies);
- Studies that explored feelings of loneliness and anxiety caused by social distancing in dementia patients and their carers (9 out of 23 studies);
- Studies that focused on family members’ fear of contagion both in-home care by informal caregivers themselves and fear of contagion when attending health services (5 of the 23 studies included); and
- Studies that focused on new forms of healthcare to address the pandemic health situation and counteract the impossibility of physical contact (12 of the 23 included studies).

### Staff shortages and the collapse of health services

One of the most essential studies providing information about staff shortages and the collapse of health services during the pandemic was published by Aker et al. in the United Kingdom, who interviewed patients and health professionals using a qualitative approach. Their results showed that one of the main concerns that remained for people with dementia and their caregivers about care was the difficulty of accessing health and social care as they used to have to request further consultations about their health situation, changes in medication, or follow-ups^12^. This was emphasized by other articles, which also reflected the significant concern of these people about the shortage of staff who carried out routine care for people with dementia, both at home and in healthcare facilities^13,14,16,17,18^. Care for people with dementia sometimes took a back seat in the pandemic health situation and healthcare collapse, in which specific diseases or health situations were considered a higher priority than dementia^18,19^.

In a review of the most current literature by Defrancesco et al., the authors added the general dissatisfaction of the dementia population and their primary caregivers about sudden changes in treatments because “it is the easiest and quickest” instead of thoroughly studying the patient’s health situation and tailoring treatment accordingly^21^. There is evidence of numerous cases of surgical interventions, in-patient treatment, and medical consultations in which the process was interrupted due to the social distancing imposed against the spread of coronavirus infection, delaying the improvement in patient health and consequent faster worsening and deterioration in people with dementia^21,22,23^.

**Table 1:** Bias analysis.

Study	Definition eligibility criteria	Variable definition	Data source	Anti-bias measures	Sample size	Qualitative variable	Statistical methods	Participants description	Statistical models	Study variables description	Confounding bias	Discussion and study limitations	Financing and conflicts
Aker et al.^12^			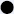		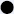			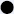	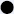				
Giebel et al.^13^			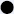		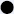				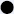			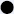	
Cuffaro et al.^14^			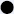		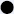				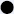				
Page et al.^15^									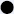				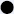
Stubbs et al.^16^			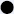		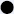				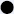				
Giebel et al.^17^	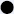		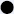		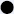		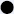	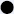	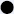			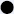	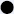
Behera et al.^18^	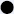	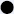	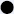		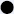		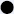	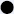	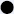	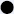			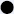
Bolt et al.^19^	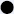		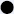		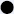		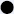	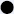	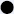	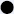		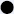	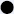
Carbone et al.^20^			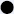		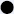	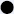		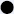	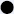				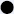
Defrancesco et al.^21^								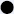	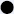				
Killen et al.^22^									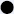			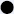	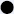
Rajagopalan et al.^23^			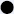		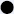	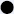	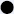	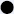	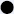			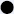	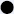
Giebel et al.^24^	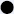		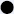		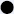		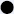	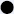	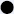			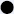	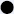
Cousins et al.^25^			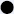		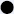				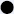				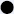
Tujit et al.^26^			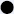		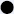			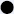	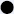			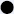	
Zucca et al.^27^			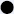		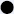			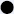	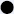				
Savla et al.^28^	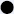	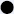					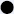	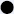	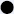				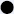
Tujit et al.^29^		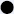	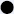		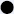			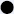	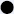			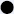	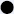
Geddes et al.^30^			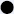		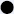				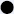			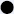	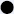
Lai et al.^31^	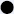		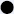		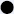	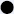			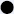	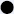			
Gosse et al.^32^			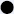					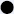	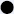				
Tousi^33^			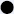		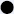				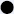				
Benaque et al.^34^		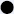	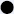		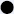	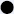		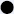	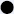				

On the other hand, a study carried out in England through interviews showed that health professionals caregivers of people with cognitive impairment were claiming a better adaptation to the pandemic situation, the inclusion in their action plans of people with dementia, and the importance of quality care for their cognitive maintenance, while also alluding to the need for greater recruitment of healthcare staff^24^. However, Cousins et al. emphasized that the origin of this understaffing and collapse is due to a governmental inability to cope with a pandemic situation in the health services and that there is no provision of the necessary infrastructure to deal with emergencies such as in this case or in a war situation where medical care is also required excessively^25^.

### The mental health of people with dementia and burnout of their carers during the COVID-19 pandemic

Nine studies addressed the feelings and psychological repercussions of people with dementia and their caregivers during the pandemic period. With the arrival of the pandemic situation, attempts were made to restrict physical contact between people as much as possible in order to curb the rate of contagion. This situation of confinement led to a feeling of loneliness and deprivation of stimuli for everyone but it was even more severe for people with particular cognitive impairment, which caused an exponential worsening of their health^21^ and high levels of stress^17,18^. Other researchers pointed in the same direction, such as Tujit et al., who described the feelings they experienced during this period of the pandemic, admitting psychological effects and negative behaviors, such as apathy, irritability, or anxiety due to the social restrictions imposed and blaming it on the lack of social engagement^26^. Rajagopalan et al.^23^ conducted a study in India that corroborated the findings of Tujit et al.^26^ regarding the negative changes seen in the behavior and cognitive status of people with dementia caused by the lack of necessary healthcare^23^.

Similar results were obtained in other countries from different cultures and continents. For example, research carried out in Italy through a survey of 4,913 informal caregivers in 2021 further added that around 20–30% of patients had depression, social isolation, and feelings of abandonment^27^, including feelings of distress after the strict confinement experienced^20^.

Much of these feelings were due to a significant lack of protection by the health system and government, noting that the services were not adapted to the health needs of patients with cognitive impairment, therefore were not able to adequately address their care, which causes them fear and hopelessness at times^24^.

On the other hand, as for the presence of burnout syndrome in informal caregivers of patients with dementia, a study by Savla et al. showed that 32% of them did not receive the necessary help or care from relatives, often because these relatives could not go to the homes where the patients lived. Some caregivers commented that they felt frustrated because, in the pandemic, they were the only ones responsible for providing the necessary care for the patients once there was no provision from the health services due to the collapse. These carers felt vulnerable to adverse psychological conditions because of role overload^28^.

Complementarily, the study by Tujit et al. added that the care previously provided by several family members, with the social distancing required during the pandemic, in many cases, only one family member carried the entire responsibility and burden of caring for the person with dementia. In addition, to avoid infecting the person for whom the care was intended, who was considered highly vulnerable to more disabling symptomatology and with a greater possibility of mortality, the family caregiver felt the additional responsibility of not interacting with too many people, which led to a sense of social isolation and loneliness^26^. At other times, unpaid caregivers were forced to make personal sacrifices in order to care for the cognitively impaired person, which also led to high levels of anxiety, helplessness, and feelings of emotional exhaustion^17,18,27^.

### Concern about possible contagion during care

Fewer studies reported the fear of being infected at home by informal caregivers or in the healthcare setting than the other variables studied.

Giebel et al. and Tujit et al. revealed that many of the caregivers interviewed expressed concern about going to the hospital or primary care health services, as they feared being infected by the virus and putting their cognitively impaired loved ones at risk^13,29^. A systematic review conducted in Canada identified that the waiting rooms of emergency departments of healthcare services were less sought after by the general population and even less by people with cognitive impairment due to the fear of possible infection once they are so prone to specific comorbidities^30^.

Moreover, caregivers sometimes feared the spread of the virus at home when requiring outside help because the person could transmit the coronavirus disease; in turn, fearing they might do so, the professionals visited people with cognitive impairment less often. It adds to the above, a feeling of loneliness and abandonment in the person with dementia^17,20,21^.

### New proposals for a healthcare approach in times of pandemic by COVID-19

Firstly, it is worth noting that almost all of these 23 articles mentioned telemedicine or healthcare by other remote means, such as the Internet (Zoom or Whatsapp), video or telephone calls, using this as a gold standard model for safety in healthcare in terms of trying to curb the rate of contagion in times of pandemic^14,17,18,20,24,26,29,30,31,32,33^.

Although telemedicine has been used as a way to bridge the healthcare gap, there were several cases of people with dementia and caregivers who did not fully agree with this solution; they sometimes admitted that they felt lost using new technologies for teleconsultation and that these methods were not adapted to the capabilities of the elderly and less affluent population^18,32^. Other studies added that the dissatisfaction of interviewees was due to their opinion that this type of care was not influential in the absence of a face-to-face link with the patient; therefore, the practitioner could not analyze holistically and comprehensively the situation surrounding the patient’s health and lifestyle, which should make it impossible to prescribe a new medication or vary medication already prescribed, recommend therapeutic activities tailored to each patient, or visualize and diagnose a physical condition correctly^24,29,33^.

However, in another study conducted in Hong Kong (China) by Lai et al., the people interviewed acknowledged that this way of dealing with the impossibility of attending an in-person consultation and receiving the care to which they were accustomed was positive for maintaining the cognitive status of dementia people, since “in situations of scarcity, better to receive remote care than no care at all”^31^. Supporting this positive image of telemedicine, an Italian study by Cuffaro et al. added that some authors revealed that remote neuropsychological assessments were beneficial in times of social restrictions, providing very accurate information about the cognitive status of patients, although not so much about the assessment of their motor and visual skills^14^.

On the other hand, other publications reported on different methods that served as a complement to telemedicine. An example of this is the crucial work of a Primary Care team of nurses in England in which they telematically sent information sheets and leaflets to people with dementia, offering, in a visual and didactic way, adapted to the cognitive level of the patient, exercises in memory, mobility, and reasoning to continue the cognitive stimulation of patients with dementia and avoid physical contact in the health services^26^.

Differently from the two previous articles, another study in this review pointed to better care for people with cognitive impairment on an ethical basis, following non-maleficence and beneficence principles, procedural justice, the dignity of a dignified death, well-being, and safety. This study defended these ethical aspects, stating that care would be effective if these principles were followed, assuring the patient a good treatment despite the new global situation of social distancing^25^.

## DISCUSSION

Overall, we identified 23 studies that discussed how the COVID-19 pandemic has influenced care for people with cognitive impairment or dementia. Among these studies, we found more information about the psycho-emotional repercussions of the pandemic on these patients and their relatives, the shortage of staff, and the collapse of health institutions due to the social and health emergency situation^12,13,14,15,16,17,18,19,20,21,22,23,24,25,26,27,28^. To a somewhat lesser extent, much information was also found about the methods of approach implemented in different parts of the world, with telemedicine being the most commonly used mode of healing^14,17,21,23,24,25,26,29,28,29,30,31,32,33,34^.

A smaller number of studies, however, commented on the fear experienced by patients and their relatives or informal caregivers when visiting health services or hiring home assistance regarding contagion and the initially unknown comorbidity of coronavirus^12,13,22,29,30^. A study by Ferretti et al. in 2021 showed the usefulness of online training for caregivers of people with cognitive impairment or dementia to detect possible physical and psychological complications, thus reducing comorbidities and contributing to a healthier life from home, without the need to physically go to the healthcare unit^35^. In terms of the psycho-emotional repercussions in a pandemic situation, none of the examples taken from previous viral outbreaks resembles the psychological impact of the new coronavirus pandemic. The extension of the pandemic and the impossibility of social interaction between family members and acquaintances has produced numerous psychological symptoms such as anxiety, depression, loneliness, and feelings of abandonment, or weakness in the face of the new situation^36^. These were added to the restriction of psychological care by health services due to the fear of contagion and the different measures taken against the spread of the virus, which forced them to prioritize other types of healthcare, leaving them exposed to all kinds of consequences of this type^37^, fact that was supported by studies such as that of Jennings et al.^38^.

On the other hand, one study reported the fear of contagion that happened during the Ebola outbreak, discussing the over-reporting of the severe clinical symptoms of the disease and how worrying and alarming it seemed to the general population, which led, as during the early part of the COVID-19 pandemic, to a decrease in the demand for healthcare in emergency departments^39^. Groothuijse et al. commented on this decrease in demand for medical care, while the vital importance of care for people with cognitive impairment during the pandemic should be emphasized as well as the increased vulnerability and emotional impairment that the pandemic has caused in this population, highlighting the requirements for a comprehensive approach to care^40^.

Finally, the methods of approach during the COVID-19 pandemic outlined in the content of this review were somewhat similar to other health emergencies. An example is the 1918 Influenza virus outbreak when the social distancing and a change in healthcare policies were chosen^41^. However, as for telemedicine, although used in some localities, it was not as widespread as during the COVID-19 pandemic, but care was rather focused on emergencies, postponing appointments, and interventions in order to avoid contact^42^. Telemedicine has proven to be a valuable resource for the care of patients with physical difficulties. It forced the population to adapt to a new form of medical assistance but this is not always achieved under a model of equality of care, once patients with low economic resources do not have the same opportunities as the rest of the population, as shown in studies such as those of Aranda et al. and Suemoto et al.^10,11^

As a concluding point of discussion, it is worth highlighting the importance of good preparation in terms of infrastructure, personnel, and resources to deal with future emergency health situations in order to cover all fronts, acute cases of the disease itself present at the time, and the assistance and care for people with chronic diseases, allowing the safeguarding of the care that is so important and necessary for these people and their families. For this, it would be advisable to have a planned and well-structured contingency plan, as well as solid approaches that allow for comprehensive care for the entire population that is adapted to the current health reality. However, despite having experienced similar situations, the political and health systems are not sufficiently prepared for the possibility of new births under such precarious health conditions.

In conclusion, the different topics addressed in this systematic review have formulated the consequences of this health situation in a broad and general way. Fourteen studies referred to staff shortages and the collapse of healthcare institutions, pointing to their great relevance in the care of people with dementia since the unavailability of qualified staff leads to a loss of quality in the care of the patients and a worsening of the clinical condition of the disease. Some of these studies even reported feelings of loneliness or isolation in a situation of global health emergency such as the pandemic. In addition, medical care and follow-up were left aside, which led to great discontent among the general population. On the one hand, the professionals were initially highly acclaimed by the population due to their apparent courage and bravery in being at the forefront of the battle against COVID-19. However, this apparent positivity was disregarded, leading to situations of hatred and despair on the part of the people treated in these health services, causing countless complaints about the lack of concern and attention given to the population.

Regarding the methods of approach to mental healthcare of patients with dementia and burnout in informal or family carers during the COVID-19 pandemic, there were numerous psycho-emotional symptoms that they experienced due to social distancing and the impossibility of relating with other people, such as feelings of loneliness and abandonment, anxiety, depression or sadness. As for the overburdening of informal caregiver or relatives, the burden of care that fell on them was still added to personal responsibilities and overall concern about the health situation, leading, most often, to an extreme feeling of anxiety, overwhelm, and emotional precariousness. These psychosocial consequences led to a mental weakening in the population, both in sick or care-seeking people, their carers, and health professionals.

As a consequence of the fear of contagion in the different forms of care provision, there was a certain reluctance to attend emergency services by patients and relatives, which sometimes led to voluntary distancing from the health services and, thus, non-attendance in situations of notable worsening in the clinical condition of the patients. This means that many conditions that could remain unnoticed or were not considered serious by the patient end up being of critical importance. There was also some fear in hiring home carers because of the possibility of contagion due to the inability to control possible contact between strange people infected with COVID-19 and their sick relatives.

Lastly, in the models for dealing with the lack of healthcare, telemedicine was found to have positive results. Most studies in this review alluded to this form of remote care, describing it as suitable for addressing health issues in patients with dementia assessing their neuropsychological evolution. However, the inability to access this type of technology and the apparent uselessness for some patients has classified telemedicine as something that should be improved in general terms, and not by case, despite a possible improvement of the pandemic situation with the use of telemedicine as a replacement for face-to-face care.

In addition to telecare and telemedicine, other methods of approaching healthcare in times of social distancing were found for patients with cognitive impairment or dementia. We can cite the didactic leaflets sent to patients’ homes to work them cognitively; new work policies in the institutions in charge of the care of these patients; or the guarantee of better care according to fundamental ethical principles such as the non-maleficence and beneficence, which have also been shown to have significant impact and benefits in caring.

Finally, it is worth highlighting the tremendous psycho-emotional impact on the part of patients and their relatives or informal carers due to social distancing, staff shortages, and healthcare collapse, which led to significant emotional deterioration and a worsening in the clinical condition of a large part of the population with dementia. In order to resolve this situation, attempts were made to offer other healthcare methods that brought good results, such as telemedicine or new healthcare policies. However, this type of solution was not suitable for a minority of the population due to specific economic, functional, or cognitive causes that make it impossible for telemedicine to be helpful as a method of addressing social distancing in healthcare.
